# Urgent dental care on a national level during the COVID‐19 epidemic

**DOI:** 10.1002/cre2.383

**Published:** 2020-12-29

**Authors:** Tadej Ostrc, Krunoslav Pavlović, Aleš Fidler

**Affiliations:** ^1^ Department of Prosthetic Dentistry, Dental Division, Medical Faculty University of Ljubljana Ljubljana Slovenia; ^2^ Department of Prosthetic Dentistry, University Dental Clinic University Medical Centre Ljubljana Ljubljana Slovenia; ^3^ Medical Chamber of Slovenia Ljubljana Slovenia; ^4^ Community Health Center Ljubljana Ljubljana Slovenia; ^5^ Department of Endodontics, Dental Division, Medical Faculty University of Ljubljana Ljubljana Slovenia; ^6^ Department of Endodontics, University Dental Clinic University Medical Center Ljubljana Ljubljana Slovenia

**Keywords:** COVID‐19, dental care, infection control, practice management, SARS‐CoV‐2

## Abstract

**Objective:**

This paper aims to report and discuss the organization and statistics of dental care during the COVID‐19 epidemics on the national level in Slovenia, providing helpful information to health policy planners worldwide.

**Material and methods:**

During an eight‐week lockdown, Emergency Dental Centers (EDCs) were established and coordinated on the national level to treat patients' urgent dental conditions. Telemedicine was used on the first level of triage to reduce contacts between healthcare workers and patients. Weekly coordination between EDCs was supported by real‐time data acquisition on the number of patient visits, prescribed medicine, the number and type of dental procedures, and the usage of personal protective equipment (PPE).

**Results:**

In EDCs, 27,468 patients were serviced, on average 235 patients per day/million people. The care was provided by 4798 man days of dental health care workers. Except for the first week, treatment and triage visits showed a slight increase. The number of incisions was nearly constant, while the number of extractions increased. A nearly threefold increase was found for emergency endodontic treatments (EET). The number of antibiotic prescriptions demonstrated an increasing trend. Analgesic prescriptions showed a decreasing trend from the beginning of lockdown.

**Conclusions:**

The reorganization and centralization of dental care proved to be an efficient model in Slovenia for the provision of urgent dental care, and the management of the healthcare workforce and PPE. Data from this study may provide helpful information for planning the needs and corresponding resources for the next waves of epidemics of COVID‐19.

## 1 INTRODUCTION

The World Health Organization declared the coronavirus disease 2019 (COVID‐19) outbreak, caused by severe acute respiratory syndrome coronavirus 2 (SARS‐CoV‐2), to be a pandemic on March 12, 2020. The COVID‐19 is associated with human‐to‐human transmission (van Doremalen et al., 2020) and, according to epidemiological data, 2019‐nCoV has higher transferability than SARS‐CoV and MERS‐CoV (Chen, 2020).

Dentistry is an integral part of medicine as there are many connections between oral and systemic conditions (Li et al., 2000). Oral diseases are among the most prevalent diseases globally and have serious health and economic burdens, greatly reducing the quality of life for those affected (Jin et al., 2016). Like other medical disciplines, dentistry has also been affected by the COVID‐19 epidemic, making it necessary to adapt considerably to the new epidemiological situation (Ather et al., 2020; Peng et al., 2020).

Regarding the exposure to SARS‐CoV‐2 aerosols and airborne particles, dental procedures could be compared to bronchoscopy or endotracheal intubation (Thamboo et al., 2020). SARS‐CoV‐2 has also been found in the saliva of infected patients (To et al., 2020). Therefore, dental healthcare workers are at high risk for SARS‐CoV‐2 infection due to the proximity of the patient's face and constant exposure to oral cavity fluids and secretions of respiratory system.

A unique characteristic of dental procedures is the extensive use of rotary and ultrasonic instruments. By this, a large amount of aerosol, droplets and splatter is created (Peng et al., 2020), and the spread of microorganisms occurs towards the dentist's face, including the mouth, nose, and eyes, which are significant for possible infection transmission (Nejatidanesh et al., 2013). Additionally, aerosol particles are small enough to remain in the air for an extended period and can travel through the air by more than 1.8 m before settling on surfaces or entering airways (Kutter et al., 2018). Concentrations high enough for infection most likely remain in the air after a cough for at least another hour or two (van Doremalen et al., 2020).

Due to the high infectious potential of dental procedures, standard protective measures in clinical work may not be effective enough to prevent the spread of COVID‐19, especially when patients are unaware that they are infected or choose to conceal the infection (Meng et al., 2020). Mainly because asymptomatic infections are possible and transmission occurs before symptoms of the disease appear (Chan et al., 2020), the change in standard precautions and the transmission regime of infections during the 2019‐nCoV epidemic is essential. Due to the high risk of infection, all non‐urgent dental treatments should be postponed. While several recommendations for the provision of dental care during epidemics have been published (Ather et al., 2020; Capocasale et al., 2020; Diegritz et al., 2020; Izzetti et al., 2020; Meng et al., 2020; Ren et al., 2020), no reports on dental service organization, provision, and needs are available.

This paper aims to report and discuss the organization and statistics of dental care during the COVID‐19 epidemics on the national level in Slovenia, providing helpful information to health policy planners.

## 2 METHODS

**FIGURE 1:**
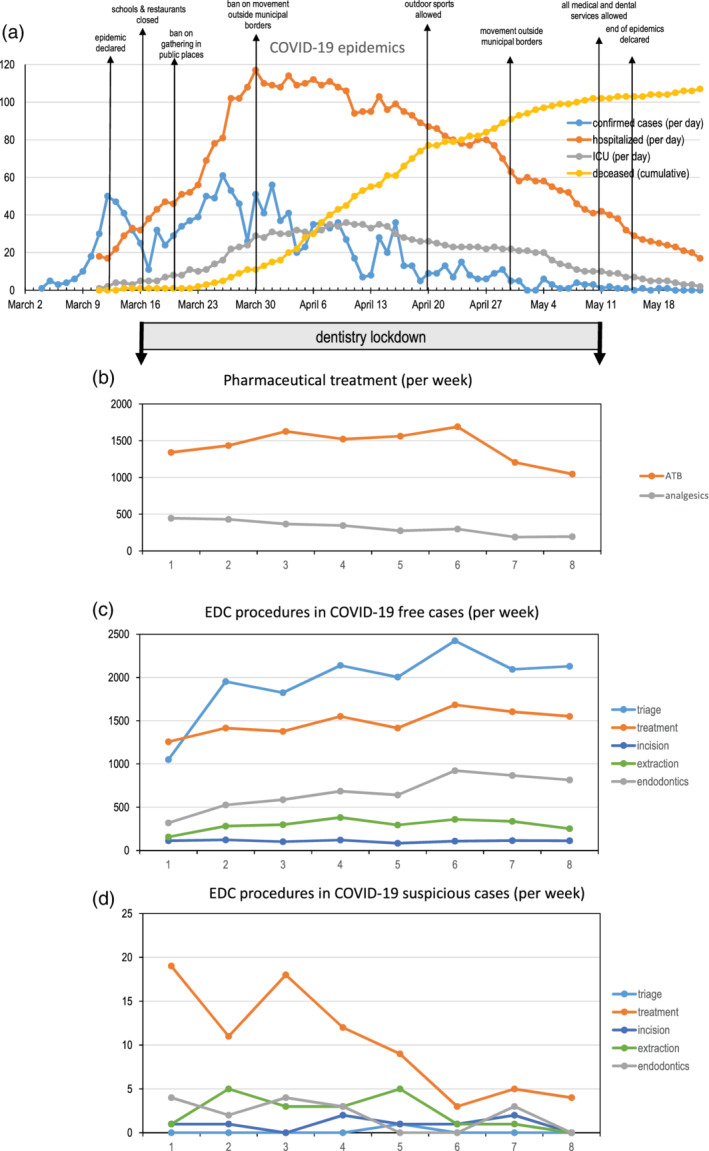
COVID‐19 situation and emergency dental procedures during the epidemic (a) Epidemic situation showing confirmed, hospitalized, intensive care unit patients (per day) and deceased (cumulative) patients since the outbreak of the contagion to the end of the epidemic; source: https://covid‐19.sledilnik.org/en/stats (b) Prescriptions of antibiotics and analgesics in dental care during lockdown (per week), the number of performed triage and procedure visits, endodontic emergency treatments, extractions, and incisions(per week) in (c) non‐suspected COVID‐19 patients, and (d) in suspected/positive COVID‐19 patients. Note that the scale of the vertical axis in the plot (d) is 100‐fold smaller in comparison to the plot (c)

The COVID‐19 pandemic spread to Slovenia on March 4, 2020, when the first case was confirmed (Figure 1a). It was an imported case of a Slovenian citizen, returning from Morocco via Italy, which was the center of the SARS‐CoV‐2 in Europe. On May 15, 2020, Slovenia became the first European nation to declare the end of the COVID‐19 epidemic within its territory (Figure 1a).

### 2.1 Slovenian dental care system

In 2018, in Slovenia (population of ca 2,095,000) there were 69.0 dentists per 100,000 inhabitants, which is slightly below the average of the EU (74.0) (Eurostat, 2020). Healthcare expenditure is comparable to that of other developed European countries (Eurostat, 2017). Dental services in Slovenia are performed on the primary level by general dentists (86%) and secondary (out‐patient care) and tertiary levels (in‐patient and out‐patient care) with dental specialists (14%) (Medical Chamber of Slovenia, 2020). Dental health services with approximately 2,400,000 visits per year for out‐patient care are offered in Community Health Centers (42%), private dental offices with contracts with the National Health Insurance (40%), and other private dental offices (18%) (Medical Chamber of Slovenia, 2020). Membership in the Medical Chamber of Slovenia (MCS) is mandatory for all practicing dentists in Slovenia.

### 2.2 Organization of dentistry services during epidemics

The Ministry of Health halted patient dental services on the primary and secondary levels of the health system in Slovenia on March 16, 2020. Emergency dental centers (EDCs), responsible for providing urgent dental care on the primary and secondary levels were instituted by Ministry of Health. The in‐patient services on the tertiary level at the two university medical centers remained opened for urgent dental care.

The MCS established a protocol for performing dental services (PPDS) during the COVID‐19 epidemic that was based on the literature available at that time (Ge et al., 2020; Meng et al., 2020; Peng et al., 2020; The Lancet, 2020; van Doremalen et al., 2020), and it was confirmed by an advisory body to the Ministry of Health for dentistry. The PPDS defined a list of urgent dental conditions, the use of PPE (see below), patient management protocols on the primary level, and a network of EDCs in Slovenia. Only patients with an urgent dental condition were treated at EDCs:

- acute, severe toothache, when analgesics are no longer sufficient,
- acute infection, presented by swelling spreading on the neck, difficult mouth opening, and raised body temperature,
- major bleeding in the oral cavity that cannot be stopped,
- dental trauma.

The level of PPE required to work at EDC for urgent dental conditions was similar as suggested for anesthesiology and emergency medical services (Centers for Disease Control and Prevention, 2020a, 2020b; European Centre for Disease Prevention and Control, 2020; Verbeek et al., 2020):

- a headwear, covering hair and ears,
- FFP2/FFP3 respirator,
- goggles/face shield,
- water‐repellent coveralls,
- disposable long‐sleeved water‐resistant surgical gown,
- footwear protection,
- double investigative gloves (certified and tested for virus transmission).

At a certain point, a protocol for reuse of PPE was prepared; due to sufficient supply, it was not activated (Center for Disease Control and Prevention, 2020).

**FIGURE 2:**
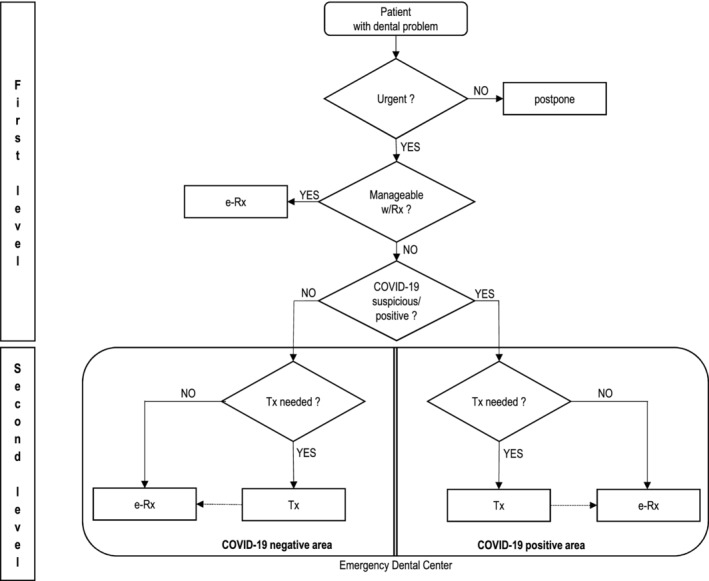
Two‐step triage and workflow of dental services during COVID‐19 epidemics in Slovenia. Triage on the first level was based on a telephone conversation and phone pictures, which were sent to a local treating dentist by e‐mail. The local treating dentist referred acute dental emergencies to the EDC. The EDC had a second triage level at its entrance. The second level triage dentist confirmed or denied the first claim of the clinical picture. EDCs had strictly separated areas for COVID‐19 positive/suspected or non‐suspected patients. All rejected patients on first or second triage received instructions on how to monitor their symptoms and chief complaint

At first, seven EDCs were established throughout the country, and two additional EDCs were established during the following 3 weeks. The patient management pathway is shown in Figure 2. Dentists in the primary level were the front line of the triage process as they attempted to resolve the condition by use of teledentistry. Real‐time consultation by phone calls and smartphone images taken by patients were used to determine the severity and the need for an urgent dental procedure. Because of the electronic prescription system (ePrescription) implemented in early 2016, most of the medication was prescribed online, without physical contact. If a dentist determined that a dental procedure was needed, the EDC was informed and the patient was referred to the EDC. Medical data (X‐rays, dental status, etc.) were delivered to EDCs by electronic devices; therefore, non‐EDC dentists could work from home. In such an emergency state, compliance with all the rules on the protection of personal data could not always be respected.

Each EDC had two separate areas based on the clinical status of patients – non‐suspected and suspected/positive COVID‐19. The separation within EDC was made to prevent the spreading of infection between patients visiting the EDC. Each EDC had one or more dental offices, operating in two daily shifts for 4 h in order to use PPE efficiently. The number of operating dental offices in each EDC was adjusted to number of patients. EDCs offered dental specialty treatment on the basis of consultation on the dentist‐specialist level and specialty services. Panoramic X‐ray services were not available in all EDCs, due to structural capacities.

Coordination of EDCs was performed by a group consisted of members of MCS, the National Institute of Public Health, the Ministry of Health, specialists‐consultants, and EDC coordinators. On average, two video conferences per week were performed, typically lasting for 2.5 h. During these meetings, EDC coordinators were able to address problems regarding PPE, patient management and organization, share experiences, get opinions from specialists, and receive psychological support. During the first conference, the need for a simple yet efficient data acquisition tool was recognized to follow the number of procedures, staff, patient demands and availability of PPE. Using Google Docs two spreadsheets were designed. One was for staff, covering the number of patients and procedures on the level of EDC. The second was for the consumption and delivery of PPE. The EDC coordinators were given access to both sheets and entered the data on a daily basis. The summary sheet, recapitulating the data in real‐time was also created, thus enabling an overview of the situation and emerging trends. The data on consumption and delivery of PPE at each EDC facilitated optimized delivery of PPE from the state reserve institution.

### 2.3 Statistical analysis

Dental service data were pooled by week and presented in time plots in relation to COVID‐19 epidemiological data to minimize the effect of daily fluctuations. Microsoft Excel 2016 (Redmond, WA, USA) was used for analysis.

## 3 RESULTS

The number of antibiotic (ATB) prescriptions demonstrated an increasing trend, peaking in the 6th week, followed by a pronounced decrease (Figure 1b). Analgesic prescriptions showed a decreasing trend from the beginning of lockdown (Figure 1b). The number of ATB prescriptions was roughly three times higher than that of analgesic prescriptions.

The descriptive statistics of staff and services at EDCs during lockdown are provided in Table 1. In non‐suspected EDC service, 27,468 patients were serviced by 4798 dental health care workers. A sharp increase of triage was found from the 1st to the 2nd week (Figure 1c). Except for the 1st week, treatment and triage showed a highly similar trend. The number of incisions was nearly constant, while the number of extractions increased, peaking in the 4th week, followed by a slight decrease. A nearly threefold increase was found for emergency endodontic treatments (EET).

**TABLE 1:** Number of staff and services at EDCs during lockdown

	COVID‐19 status							
	Total		Per week		Per day		Per day/1.000.000	
	−	+	−	+	−	+	−	+
Nurses	2734.00	328.00	341.75	41.00	48.82	5.86	23.30	2.80
Dentists	2064.00	168.00	258.00	21.00	36.86	3.00	17.59	1.43
Triage visits	15,617.00	1.00	1952.13	0.13	278.88	0.02	133.11	0.01
Tx	11,851.00	81.00	1481.38	10.13	211.63	1.45	101.01	0.69
Incisions	875.00	8.00	109.38	1.00	15.63	0.14	7.46	0.07
Extractions	2357.00	19.00	294.63	2.38	42.09	0.34	20.09	0.16
EET	5364.00	16.00	670.50	2.00	95.79	0.29	45.72	0.14
Specialty consultation	101.00	0.00	12.63	0.00	1.80	0.00	0.86	0.00
Specialty procedures	87.00	1.00	10.88	0.13	1.55	0.02	0.74	0.01

The number of patients suspected of being infected represented a small fraction (approximately 1/300) of the non‐suspected patients (Table 1). The number of triages was decreasing with time, while the number of extractions, incisions, and EET remained constant (Figure 1d).

## 4 DISCUSSION

The described organization exhibited adequate urgent dental care, indicated by a non‐increasing number of provided treatments accompanied by efficient infection control and the efficient use of PPE. In non‐epidemic situation on average 4590 patients per day/million are served in Slovenia. 235 patients per day/million people represent a number of people, seeking urgent dental treatment in epidemic situation during lockdown. Comparison to other countries is not possible, as no similar study has been published so far. During the lockdown, the number of operating dentists in EDCs on average was 18 per day, representing approximately 1.5% of the operating dentists in non‐epidemic conditions. These results also offer insight into urgent dental treatment needs, with 133.11 triage visits and 101.01 procedures performed daily per 1 million people.

A considerable part of dental care was performed exclusively with teledentistry at the first line of the triage, thus reducing both the patient load at the EDCs and unnecessary social contact. Although the increasing importance of telemedicine has been recognized, it has become increasingly important during the COVID‐19 pandemic (Hollander & Carr, 2020), as it can assist in remote triage and continuity of care, both for those infected and not infected with COVID‐19, reducing the risk for the spread of infection.

From the results, it is shown that the number of dental procedures in cases when the patient was suspected of being infected was relatively small in comparison to the non‐suspected cases. Those were the cases that could not be postponed or relieved by the use of medication. Trends of treatments show that the number of incisions remained relatively constant. The decreased number of prescriptions could be explained by a larger number of extractions and EET performed over time, reducing the need for pharmacological treatment. The low number of prescribed analgesics in comparison to ATB could be attributed to self‐medication possible with over‐the‐counter availability of acetaminophen and ibuprofen.

The amount of aerosol in dentistry considerably depends on the type of procedure. A draining incision is usually a quick procedure, compared to an extraction, and both are performed without rotary instruments. In contrast, EET is more time consuming and performed with rotary instruments, which generate aerosol. The increased number of more complex and more aerosol‐generating procedures over time could be explained by the fear reduction and increased confidence (Pfefferbaum & North, 2020) as the COVID‐19 pandemic has caused considerable concerns to dental practitioners worldwide (Nibali et al., 2020).

Based on analysis of our data we can conclude that the system was designed appropriately and that the needs were assessed correctly, as there was no uncontrolled burden on EDCs or an increase in the number of dental services provided over time. Although our model of organization was found to be effective, it should be noted that application to other countries may require adjustment due to differences in the oral health of the population.

According to available data from the National Institute of Public Health, three dental health care workers were infected before the establishment, and none during the operating of EDCs (based on reports of EDC coordinators), which indicates good infection control and an adequate level of protection. In Italy, more than 24,000 health care workers tested positive for SARS‐CoV‐2, representing more than 10% of that nation‐s COVID‐19 cases (Istituto Superiore di Sanità, 2020). In other sectors of the health system in Slovenia, more than 160 healthcare workers were infected (however not all due to contacts at work) (https://covid‐19.sledilnik.org/sl/stats). Due to the obligation to work during epidemics, medical staff expressed physical and mental exhaustion, difficult triage decisions, and mental pain due to the loss of patients and co‐workers, in addition to the risk of infection itself (The Lancet, 2020).

The rapid establishment of EDCs was possible due to the organization of the health care system (a large number of Community Health Centers) in Slovenia, and the quick response of all involved participants. PPDS were proposed by MCS and revised and confirmed by an advisory body to the Ministry of Health for dentistry, which enabled implementation of the protocol throughout the country within one weekend. Such a short response time minimized the possible spread of infection in the early phases of the epidemic. Centralized management with a few key people and biweekly online meetings allowed a good overview of the situation and patients' needs in real‐time and better compliance with PPE instruction modification (often changing during early phases of epidemics). Efficient instructions for patients with a press conference on national television programs were possible as all EDCs were organized in the same manner. Centrally coordinated PPE management and distribution between EDCs facilitate efficient use and delivery and prevent the shortage of PPE during limited availability.

Such an organization of dental care during epidemics also has some disadvantages. Firstly, it is less accessible for patients as dental services could not be performed at their personal dentist's office. There was a large number of dental health care providers working at EDCs resulting in a not completely standardized treatment approach. There was also a large number of shifts at the dental offices for COVID ‐19 suspected cases in regard to a small number of performed dental procedures. This could raise a question of rational use of PPE and the possible spread of infection in the case of an infected dental health care provider at EDC. Another concern is the financial implications for dental practices and cost of epidemic prevention during the lockdown as the offices were forced to stop working (Schwendicke et al., 2020). It must be emphasized that epidemic data from neighboring countries (especially Italy) was concerning (Nacoti et al., 2020) and there was no guarantee that the same scenario could not also happen in our country.

Our study is the first description and analysis of dental care at the country level during an epidemic lockdown. A data acquisition protocol had to be organized in a limited time and without any prior references that could serve as a model. A disadvantage was that in the treatment process and in the collection of data, a large number of dental health care workers participated, which might affect the consistency of the data. However, this was a real‐time study and, therefore, data collection was not standardized, which is an approach of the big data concept (Obermeyer & Emanuel, 2016). Correlation and analysis of dental services and treatments of each urgent dental condition would add to the value of the study and should be used in the future studies.

## 5 CONCLUSIONS

In conclusion, the centralized system coordination, the use of teledentistry, and frequent online meetings resulted in effective and safe urgent dental care during COVID‐19 epidemics, minimizing the chances of dental offices to become the potential spread of infection (Meng et al., 2020). Data from this study may provide valuable information for planning the needs and corresponding resources of PPE at the second wave of epidemics of COVID‐19 on the national level and also is a source of information for other countries.

## CONFLICT OF INTEREST

There is no conflict of interest.

## AUTHOR CONTRIBUTIONS

All authors contributed to conception, design, data acquisition and interpretation, drafted and critically revised the manuscript. Aleš Fidler contributed also to the statistical analysis. All authors gave their final approval and agree to be accountable for all aspects of the work.

## Data Availability

The data that support the findings of this study are available from the corresponding author on reasonable request. Participant data without names and identifiers will be made available after approval from the corresponding author and National Health Commission. The proposal with detailed description of study objectives and statistical analysis plan will be needed for evaluation of the reasonability to request for our data. The corresponding author and National Health Commission will make a decision based on these materials.
